# Research on High-Precision Localization Method of Curved Surface Feature Points Based on RGB-D Data Fusion

**DOI:** 10.3390/s26010137

**Published:** 2025-12-25

**Authors:** Enguo Wang, Rui Zou, Chengzhi Su

**Affiliations:** 1 School of Mechanical and Electrical Engineering, Changchun University of Science and Technology, Changchun 130022, China; zourui@mails.cust.edu.cn; 2 School of Artificial Intelligence, Changchun University of Science and Technology, Changchun 130022, China; suchengzhi@cust.edu.cn

**Keywords:** depth cameras, sub-pixel level positioning, cross-modal fusion, curved surface feature points

## Abstract

Although RGB images contain rich details, they lack 3D depth information. Depth data, while providing spatial positioning, is often affected by noise and suffers from sparsity or missing data at key feature points, leading to low accuracy and high computational complexity in traditional visual localization. To address this, this paper proposes a high-precision, sub-pixel-level localization method for workpiece feature points based on RGB-D data fusion. The method specifically targets two types of localization objects: planar corner keypoints and sharp-corner keypoints. It employs the YOLOv10 model combined with a Background Misdetection Filtering Module (BMFM) to classify and identify feature points in RGB images. An improved Prewitt operator (using 5 × 5 convolution kernels in 8 directions) and sub-pixel refinement techniques are utilized to enhance 2D localization accuracy. The 2D feature boundaries are then mapped into 3D point cloud space based on camera extrinsic parameters. After coarse error detection in the point cloud and local quadric surface fitting, 3D localization is achieved by intersecting spatial rays with the fitted surfaces. Experimental results demonstrate that the proposed method achieves a mean absolute error (MAE) of 0.17 mm for localizing flat, free-form, and grooved components, with a maximum error of less than 0.22 mm, meeting the requirements of high-precision industrial applications such as precision manufacturing and quality inspection.

## 1. Introduction

RGB-D object detection is one of the fundamental tasks in the field of computer vision [1], with a research history of over 20 years in the academic community. Computer vision integrated with artificial intelligence is being incorporated into numerous industrial processes, and the increasing accessibility of depth data has further expanded the application scope of computer vision in quality assurance and decision-making fields. RGB-D data is being applied in scenarios such as bridge inspection [2], railway quality assurance [3], agriculture [4,5], and robotics [6]. By combining RGB data with depth data, in addition to collecting texture- and color-based information, shape-based information can also be generated, thereby producing RGB-D data [7]. Such data enables a deeper understanding of target objects, which in turn provides accurate data support for determining the key point localization of object feature points and offers additional valid references for subsequent imaging decision-making processes. Therefore, RGB-D data plays a crucial role in these scenarios. For instance, localization errors of key features such as the curved surface contour of aero-engine blades [8] and the cavity edges of automotive molds directly affect the localization accuracy of workpieces.

In the field of industrial 3D visual positioning, technical approaches for acquiring object spatial information are primarily divided into two categories: vision-based reconstruction and direct measurement using depth sensors. Among vision-based methods, while a single monocular RGB image can provide rich texture and feature point information, its imaging principle inherently lacks physical scale, making it unable to directly obtain 3D coordinates of the measured object. In contrast, multi-view (e.g., stereo) vision-based techniques, which simulate human binocular vision using multiple CCD cameras and compute depth from multi-frame images via triangulation, have matured into a cost-effective 3D reconstruction solution and have achieved success in ideally conditioned applications.

However, when targeting the specific task of high-precision (typically requiring sub-millimeter accuracy) and highly reliable localization of feature points on workpiece surfaces, stereo vision methods face inherent challenges:

- System accuracy heavily relies on complex and precise camera calibration. Factors such as temperature fluctuations and vibrations in the field environment can lead to calibration parameter drift, introducing systemic errors.
- The accuracy of stereo matching significantly degrades in areas with weak textures, repetitive textures, or strong optical reflections on smooth surfaces. This often results in depth calculation failures or substantial noise at critical workpiece feature points (e.g., intersection points of edges, corners).
- The entire processing pipeline (including image rectification, dense matching, and 3D reconstruction) is computationally intensive, making it difficult to meet the demands for online, real-time inspection cycles in industrial settings.

In comparison, RGB-D cameras, as sensors capable of synchronously and directly acquiring registered color images and depth maps (point clouds), offer an alternative efficient solution. Although depth data itself suffers from issues like noise interference and lack of detail, its “direct acquisition” nature avoids the complex reconstruction calculations and matching uncertainties inherent in stereo vision. Therefore, the focus of this research is on how to deeply integrate the sub-pixel feature recognition capability of RGB images with the direct spatial geometric information provided by depth data to construct a localization framework that eliminates the need for complex multi-camera calibration, is insensitive to workpiece surface texture, and meets the requirements for both high precision and high real-time performance. This RGB-D multi-modal data fusion technology, by bridging the rich semantics of 2D vision with the direct geometric measurement of 3D space, provides a new technical pathway for achieving stable and precise localization of feature points on workpieces in challenging industrial scenarios.

To address the aforementioned issues, this paper proposes a high-precision localization method for surface feature points based on RGB-D data fusion. Through a full-process collaborative mechanism of “feature classification–sub-pixel feature boundary extraction–3D mapping and partitioning–surface fitting–intersection point fine localization”, accurate localization of complex surface feature points is achieved. This method not only provides technical support for the high-precision feature recognition of complex components in industrial inspection but also explores a new solution for the in-depth application of RGB-D fusion technology in the field of precision manufacturing. Its main contributions are as follows:

- Clearly define two types of key feature points (angular surface key points formed by the intersection of two edges and spatial cusp key points formed by the intersection of three edges), providing a clear geometric representation basis for the accurate localization of complex surface features.
- Propose a two-stage localization framework that integrates RGB and depth information: in the coarse localization stage, the YOLOv10 model is used to realize the classification and recognition of feature points, and an additional Background Misdetection Filtering Module (BMFM) is added to reduce background interference; in the fine localization stage, an improved Prewitt operator (extended to an 8-direction 5 × 5 convolution kernel) is adopted to extract feature boundaries, and sub-pixel subdivision technology is combined to calculate boundary intersection points, significantly improving the localization accuracy of 2D feature points.
- Construct a high-precision spatial localization mechanism for “RGB-depth” cross-modal fusion: accurately map the feature boundaries extracted from 2D images to the 3D point cloud space based on camera external parameters; segment the point cloud of the target area through coarse localization, perform 3D surface fitting after removing abnormal points using a gross error detection mechanism, and finally realize 3D localization by calculating the intersection between the spatial ray corresponding to RGB feature points and the fitted surface. This breaks through the limitations of traditional methods, such as loose correlation between 2D and 3D features and insufficient robustness of surface fitting.

## 2. Related Works

Due to key points being occluded, the presence of outliers, and measurement noise, the available information is limited and ambiguous, making it quite challenging to locate the missing parts of point clouds. Workpiece localization methods can be categorized into the following types:

- Vision-based localization methods;
- Point cloud-based localization methods;
- RGB-D fusion-based localization methods.

### 2.1. Vision-Based Localization

Traditional 2D vision methods rely on edge detection (e.g., the Sobel [9] and Prewitt operators, which are suitable for horizontal and vertical edge detection; the Canny [10] operator improves edge continuity through double-threshold filtering, though its noise resistance depends on parameter tuning) and corner extraction (e.g., the Harris corner detector [11]) to achieve localization. However, these methods can only acquire 2D image information and lack depth data, making it difficult to reflect the real pose and positional relationship of workpieces in 3D space. As a result, their application in 3D industrial scenarios is limited.

Deep learning-based methods (e.g., the YOLO [12] and Faster R-CNN [13] series) perform well in target detection of industrial components, enabling rapid identification of target regions. Nevertheless, their localization accuracy is usually confined to the pixel level. Additionally, due to the scarcity of dedicated datasets for industrial components, these methods exhibit insufficient sub-pixel-level classification capability for complex composite features (such as multi-edge intersection points) in complex scenarios, which hinders their support for subsequent high-precision differentiated localization strategies. Furthermore, although deep learning-based edge detection methods (e.g., HED networks) can extract semantic edges [14], they incur high computational costs and struggle to meet the real-time requirements of industrial applications, limiting their use in dynamic localization scenarios.

Furthermore, localization methods based on binocular or multi-view stereo vision, which recover the three-dimensional structure of a scene from multi-view RGB images through epipolar geometry and dense stereo matching [15], represent classical approaches in the field of computer vision. These methods offer the advantage of not relying on specialized depth sensors, thereby providing benefits in terms of system cost. However, within the context of high-precision industrial localization, their performance is constrained by several bottlenecks. Firstly, the accuracy of the 3D coordinates of feature points is jointly determined by the sub-pixel accuracy of stereo matching, the precision of camera calibration, and the baseline length. This results in a long error chain, making it challenging to consistently maintain sub-millimeter localization accuracy under complex working conditions. Secondly, as mentioned in the Introduction, in common weak-texture regions on workpieces, matching algorithms struggle to find accurate correspondences, leading to the loss of depth information for feature points.

### 2.2. Point Cloud-Based Localization

Among point cloud-based localization methods, the ICP algorithm and its variants (e.g., TrICP) achieve global registration by iteratively optimizing the transformation matrix between point clouds [16]. Their core objective is to align the global poses of two point clouds, rather than directly outputting the 3D coordinates of specific key feature points of workpieces (e.g., angular surface key points, spatial cusp key points). These methods have high requirements for the alignment accuracy of initial data. Moreover, at the key feature points of workpieces, where point clouds are sparse or missing due to depth camera noise interference or imaging angle limitations, it is not only difficult to accurately capture the spatial information of feature points but also impossible to meet the requirements of sub-pixel-level fine localization.

### 2.3. RGB-D Fusion-Based Methods

In recent years, RGB-D fusion-based localization methods have become an active research focus due to their ability to leverage both 2D textural information and 3D geometric structures [17]. While existing approaches, ranging from using 2D features to guide point cloud segmentation [18] to refining 3D coordinates via sub-pixel localization techniques, have made progress, they often suffer from limitations such as inadequate fitting capability for complex curves and insufficient consideration of edge direction diversity. Although emerging Transformer-based methods demonstrate powerful RGB-D feature fusion capabilities [19], their potential is often limited in data-scarce industrial scenarios, and they struggle to guarantee sub-pixel geometric accuracy. Consequently, a research gap remains in the sub-pixel-level key point localization for industrial workpieces. There is a pressing need to develop a robust RGB-D fusion framework that is both data-efficient and geometrically constrained, which constitutes the core objective of this research.

## 3. The Proposed Methodology

Analysis of existing technologies shows that, in industrial scenarios, the nonlinear morphology of complex surfaces, coupled with issues such as noise interference, sparse point clouds, or missing point clouds that easily occur in depth data at key feature points, makes it difficult for localization methods relying solely on point cloud data to accurately capture feature point information [20]. Meanwhile, although a single RGB image can provide rich texture details, it cannot directly obtain 3D spatial coordinates, making it hard to meet the requirements of high-precision localization. Additionally, due to significant individual differences in complex surfaces, learning-based methods require a large number of training samples, which is also difficult to achieve in industrial inspection [21]. To address these challenges, this paper proposes an optimized method based on RGB-D fusion. Unlike stereo vision solutions that rely on multi-frame images and complex calibration, our approach leverages the inherent capability of RGB-D cameras to synchronously and directly acquire registered multi-modal data, establishing a more streamlined technical pathway. Its core advantage lies in obtaining both RGB images for high-precision feature recognition and depth maps providing direct spatial constraints through a single capture. This effectively avoids the computationally intensive processes and matching failure risks inherent in stereo vision, thereby laying the foundation for achieving rapid and high-precision localization of complex curved surface feature points. The workpiece localization method includes the following key steps:

- Coarse localization
  - Establish a local feature detection dataset for components, use the YOLOv10 model to classify and identify feature points in RGB images, and add a Background Misdetection Filtering Module (BMFM) to reduce background interference;
  - Adopt an improved Prewitt operator (extended to an 8-direction 5 × 5 convolution kernel) to extract feature boundaries in RGB images, and combine binocular vision depth information to filter out background interference, thereby completing the decomposition of foreground contours;
- Fine localization
  - Fit feature boundaries through polynomials, and combine sub-pixel subdivision technology to calculate the intersection points of feature boundaries (the intersection of two edges is solved by directly combining equations, and the intersection of three edges is optimized through mean processing) to obtain sub-pixel-level key points;
  - Establish the external parameter mapping relationship between RGB and depth data based on the principle of binocular vision, and realize the conversion between pixel coordinates and 3D coordinates through the internal and external parameters of the camera;
  - Perform 3D surface fitting on the point cloud of the target area segmented from the depth image, remove abnormal points through a gross error detection mechanism, and then calculate the intersection between the spatial ray corresponding to the RGB feature point and the fitted surface to complete spatial localization.

By fusing the 2D information of RGB images and the spatial geometric information of depth data, this method constructs a complete technical chain from 2D feature detection to 3D localization. Combined with strategies such as sub-pixel subdivision and robust fitting, it breaks through the limitations of traditional methods, such as loose correlation between 2D and 3D features and insufficient robustness of surface fitting, and realizes high-precision localization of feature points on complex surfaces.

### 3.1. Establishment of RGB Feature Dataset and Improved YOLOv10 Post-Processing Method

To achieve fast and accurate coarse localization of feature regions, a high-performance object detection model is required. YOLOv10, as the latest official version of the YOLO series available at the commencement of this study (early 2024), was selected due to its state-of-the-art performance in balancing precision and speed, achieved through innovations like its NMS-free design. It is crucial to note that the overall cross-modal fusion and localization framework proposed in this paper is modular by design. The core contribution lies in the subsequent sub-pixel refinement and cross-modal precise localization mechanisms, rather than in the specific choice of the detector. YOLOv10 is employed here as an efficient and reliable component, and the framework maintains the flexibility to integrate more advanced detectors in the future.

When constructing a dataset, the first step is to define the characteristic regions of the data. From a geometric perspective, by focusing on the core structure and key morphological markers of an object, most workpiece features can be adequately described using just two types of characteristic regions: intersections of two edges and intersections of three edges.

When we concentrate on the structural characteristics of a workpiece, these two types of intersections are sufficient to form a coherent understanding of its morphology. The distribution of intersections of two edges allows us to infer the fundamental planar framework of the workpiece, while the positions of intersections of three edges directly reflect complex spatial connections in three dimensions. Therefore, these two types of characteristic regions can be regarded as the foundation for constructing the features of most workpieces, enabling an effective description of their core characteristics.

**Figure 1:**
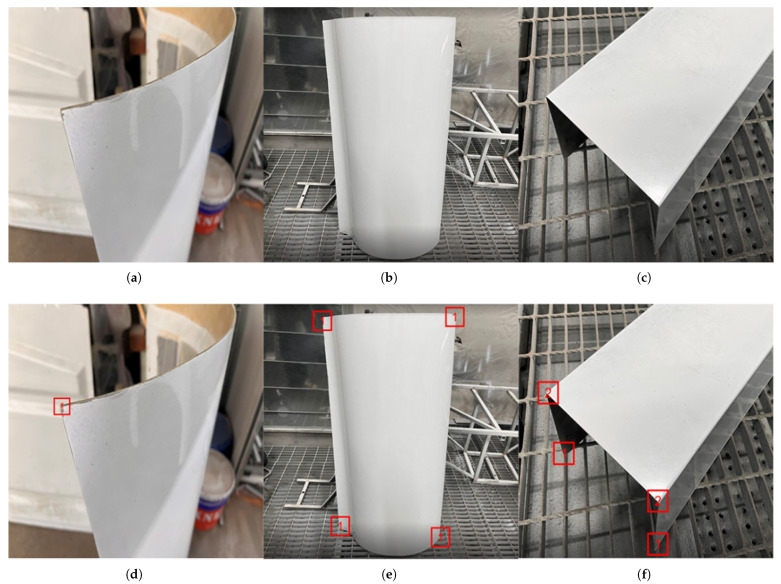
Examples of Dataset and Annotations. (**a**–**c**) Original Images; (**d**–**f**) Annotation Results.

Based on the above definitions of feature regions, this study constructs a dataset specifically focused on workpiece feature points. The original data were collected through multi-angle and multi-background imaging of three types of components: flat plates, free-form surfaces, and grooved parts. The detailed distribution is shown in Table 1. To enhance model robustness and prevent overfitting, after splitting all original data into training, validation, and test sets in a 7:1.5:1.5 ratio, we applied a data augmentation pipeline exclusively to the training set. This pipeline included random rotation (±10°), scale variation (0.9–1.1×), and color jittering to simulate realistic imaging variations. Ultimately, we established a dataset comprising 9227 images, with its detailed composition presented in Table 2. Examples of the dataset and corresponding annotations are shown in Figure 1.

**Figure 2:**
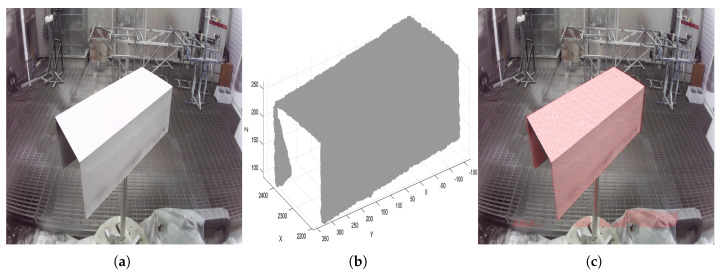
Schematic diagram of background false detection filtering. (**a**) Color image; (**b**) 3D point cloud; (**c**) Image-point cloud mapping relationship.

In unstructured complex environments, image background regions can significantly interfere with YOLOv10 detection, leading to false detections in these areas. To address this issue, this paper introduces a Background Misdetection Filtering Module (BMFM) during the model’s post-processing stage. This module leverages binocular vision technology. When a binocular camera captures target information, it simultaneously acquires both a color image (as shown in Figure 2a) and a 3D point cloud (as shown in Figure 2b). A correspondence between the two can be established using the camera’s intrinsic and extrinsic parameters. However, due to the resolution limits of the binocular camera and the disparity range between the left and right images, matching corresponding points for distant targets becomes difficult, resulting in an inability to calculate depth information for these areas. By projecting the 3D point cloud onto the color image, the result shown in Figure 2c is obtained. Here, the red areas represent regions with valid depth information, which correspond precisely to the foreground targets. In contrast, the image background lacks depth information. Based on this principle, the proposed method iterates through all feature regions detected by the YOLOv10 model and checks for the presence of depth information within each region. Regions containing depth information are confirmed as valid foreground target features and retained, while those lacking depth information are identified as background false detections and subsequently filtered out.

### 3.2. Image Foreground Contour Extraction and Decomposition Algorithm Based on Improved Prewitt Operator

The trained YOLOv10 model can detect the regions where feature points are located in the target image, achieving coarse localization of image feature points. To accurately extract the pixel coordinates of feature points, this section focuses on detecting and decomposing the target foreground contours in the feature regions. Wang Enguo et al. proposed discretizing image gradient directions into 8 directions [22] (0,π/4,π/2,3π/4,π,5π/4,6π/4,7π/4), which provides ideas for multi-directional gradient extraction. Since the Prewitt operator mainly detects horizontal and vertical edges but has a weak response to oblique edges, this paper expands the two-directional templates of the Prewitt operator into eight-directional templates. To improve anti-noise capability and achieve better smoothing effects, the original 3×3 convolution kernel is extended to a 5×5 convolution kernel. The specific convolution kernel parameters for the eight directions are detailed in Table 3.

Let the gradient operator templates in 8 directions be $T_{\alpha }$, where α is the direction index, and the 5×5 window image of the pixel (x,y) in the image be $f(x^{\prime },y^{\prime })$. Then, the gradient values $G_{\alpha }\left(x,y\right)$ of the pixel in 8 directions can be calculated using Formula (1), where * denotes the convolution operation.

(1) $$G_{\alpha }\left(x,y\right)=T_{\alpha }*f\left(x^{\prime },y^{\prime }\right)$$

Non-maximum suppression is applied to the obtained gradient values in 8 directions, and the maximum gradient value is selected as the gradient value of the point. Let the gradient value at pixel (x,y) be G(x,y) and the gradient direction be φ(x,y); then,

(2) $$G\left(x,y\right)=\mathrm{MAX}(G_{\alpha }\left(x,y\right)),\;\alpha =1,\;2,\ldots ,\;8$$

(3) $$\varphi \left(x,y\right)=\{\alpha |G\left(x,y\right)=\mathrm{MAX}\left(G_{\alpha }\left(x,y\right)\right)\}$$

**Figure 3:**
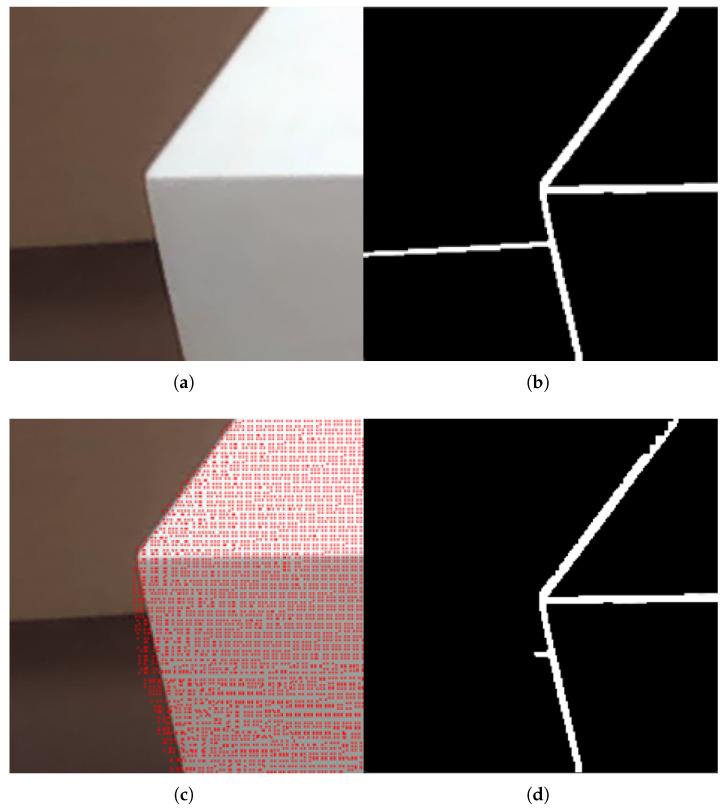
Schematic Diagram of Foreground Contour Extraction. (**a**) Component image; (**b**) Image contour; (**c**) Point cloud mapped image; (**d**) Image foreground contour.

By traversing the image to calculate the gradient value of each pixel and comparing it with the set threshold, we can determine whether the point is an edge pixel, thereby realizing image contour extraction. To filter out the interference of background information in the image, this paper combines binocular vision depth information to extract foreground contours based on the mapping relationship between point clouds and images. The result of mapping point clouds to the image is shown in Figure 3c, and the foreground contour extraction result is shown in Figure 3d.

After extracting the target foreground contour, the contour is decomposed using 8 gradient directions, resulting in 8 sub-images containing sub-contours. Let the gradient value at pixel (x,y) be G(x,y) and the gradient direction be φ(x,y). Let the pixel values of the 8 sub-images at pixel (x,y) be $G_{k}\left(x,y\right)$ (where k=1, 2,…, 8).

Traverse the foreground contour image: when φ(x,y)=0, $G_{1}\left(x,y\right)=G\left(x,y\right)$; when φ(x,y)=π/4, $G_{2}\left(x,y\right)=G\left(x,y\right)$; when φ(x,y)=π/2, $G_{3}\left(x,y\right)=G\left(x,y\right)$; when φ(x,y)=3π/4, $G_{4}\left(x,y\right)=G\left(x,y\right)$.

**Figure 4:**
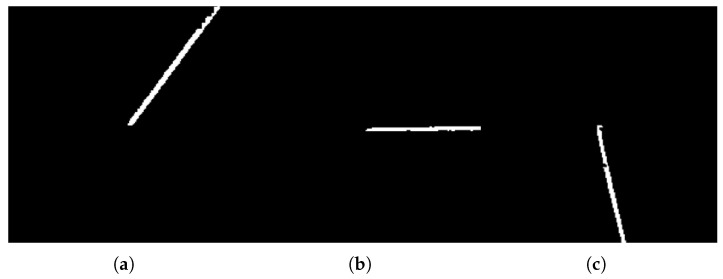
Schematic Diagram of Sub-Edge Decomposition. (**a**) Sub-edge 1; (**b**) Sub-edge 2; (**c**) Sub-edge 3.

Continue this process to complete the pixel assignment for all 8 sub-images. Then, extract continuous edges from the 8 sub-images based on pixel continuity. Decomposing the contour image shown in Figure 3d yields the sub-edges shown in Figure 4.

### 3.3. Sub-Pixel Localization Method for Feature Points in RGB Images

After obtaining the pixel points of contour sub-edges, the edge equation can be derived through polynomial fitting, which is used to solve the coordinates of edge intersection points or ellipse center points. Given edge pixel points $\left(x_{1},y_{1}\right),\left(x_{2},y_{2}\right),\ldots ,\left(x_{n},y_{n}\right)$, the goal of polynomial fitting is to find an appropriate polynomial function f(x) that can pass through these data points as accurately as possible. Considering that the edges of industrial workpieces are mostly straight lines or gently curved lines, second-order polynomials are sufficient to capture their geometric features. Furthermore, they offer advantages such as high computational efficiency, good numerical stability, and reduced risk of overfitting. Therefore, this paper adopts second-order polynomials for fitting, with the expression shown in Formula (4).

(4) $$\begin{array}{c} f\left(x\right)=a_{0}+a_{1}x+a_{2}x^{2} \end{array}$$

where f(x) is the fitting function, and $a_{0},a_{1},a_{2}$ are parameters to be solved through the fitting process. The solution for image feature points extracted by the method in this paper mainly includes the following two cases: the intersection of two edge lines and the intersection of three edge lines. First, for solving the coordinates of the intersection point of two edge lines, the expressions of the two edge lines can be obtained through polynomial fitting, as shown in Formula (5):

(5) $$\left\{\begin{array}{c} \begin{array}{c} f_{1}\left(x\right)=a_{1,0}+a_{1,1}x+a_{1,2}x^{2} \end{array}\\ \begin{array}{c} f_{2}\left(x\right)=a_{2,0}+a_{2,1}x+a_{2,2}x^{2} \end{array} \end{array}\right.$$

By solving the equations simultaneously, the intersection coordinates $\left(x_{c},y_{c}\right)$ of the two edge lines can be obtained. For solving the intersection coordinates of three edge lines, first, the polynomial expressions of the three edge lines can be derived through polynomial fitting, as shown in Formula (6).

(6) $$\left\{\begin{array}{c} \begin{array}{c} f_{1}\left(x\right)=a_{1,0}+a_{1,1}x+a_{1,2}x^{2} \end{array}\\ \begin{array}{c} f_{2}\left(x\right)=a_{2,0}+a_{2,1}x+a_{2,2}x^{2} \end{array}\\ \begin{array}{c} f_{3}\left(x\right)=a_{3,0}+a_{3,1}x+a_{3,2}x^{2} \end{array} \end{array}\right.$$

By solving the equations simultaneously, three sets of intersection coordinates $\left(x_{1,2},y_{1,2}\right)$, $\left(x_{1,3},y_{1,3}\right)$, and $\left(x_{2,3},y_{2,3}\right)$ can be obtained. To reduce calculation errors and improve robustness, although the three edge lines should theoretically intersect at the same point, deviations may exist among the three sets of intersections due to noise or edge extraction errors during the actual fitting process. Therefore, optimization is performed through mean processing: in this paper, the mean value of the three sets of intersection coordinates is calculated to obtain the final feature point coordinates $\left(x_{c},y_{c}\right)$, as shown in Formula (7).

(7) $$x_{c}=\frac{x_{1,2}+x_{1,3}+x_{2,3}}{3},\;y_{c}=\frac{y_{1,2}+y_{1,3}+y_{2,3}}{3}$$

For sub-pixel level feature points, the camera intrinsic parameter matrix is used to project them into the camera coordinate system, generating a ray equation originating from the optical center of the color camera. In the camera coordinate system, this ray can be expressed as:

(8) $$r\left(t\right)=O_{c}+t\cdot d,\;t\geq 0$$

**Figure 5:**
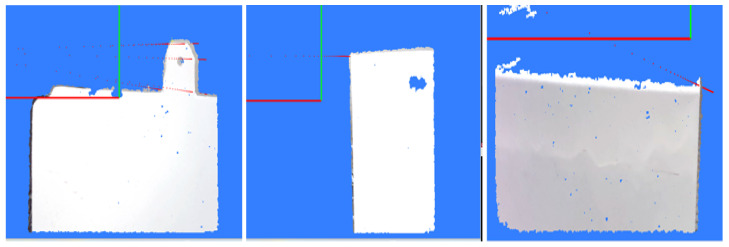
Results of Ray Calculation.

Subsequently, the camera’s extrinsic parameters are used to transform the imaging ray of the sub-pixel feature point in the color camera to the point cloud coordinate system for display, obtaining an RGB ray based on the imaging principle of the color camera, which realizes the spatial alignment of multi-sensor data, as shown in Figure 5. The transformed ray equation is:

(9) $$R^{\prime }\left(t\right)=R\cdot r\left(t\right)+t,\;t\geq 0$$

### 3.4. Establishment of Mapping Relationship Between Image and Point Cloud Based on Binocular Vision

In a binocular vision system, depth information of the scene can be calculated using stereo matching algorithms based on the two views acquired by the left and right cameras. This enables the acquisition of 3D point cloud data corresponding to the images. This section will elaborate on the process of establishing the mapping relationship between pixel points and point cloud points.

In a binocular vision system, the left camera is usually used as the reference coordinate system to define the pixel coordinate system and the camera coordinate system. The pixel coordinate system uses pixels as the unit and can be expressed as (u,v), while the camera coordinate system uses 3D space as the unit and can be expressed as $\left(X_{c},Y_{c},Z_{c}\right)$. For the matched points in the left and right images of the binocular vision system, the relationship between the disparity *d* and the depth $Z_{c}$ can be expressed using the triangulation principle as follows:

(10) $$Z_{c}=\frac{f*B}{d}$$

Among them, *f* denotes the focal length of the camera, *B* denotes the baseline distance of the binocular camera, and *d* denotes the horizontal pixel difference between corresponding points in the left and right images. By calculating the disparity of each pixel point, the depth information $Z_{c}$ of the scene can be obtained. The camera intrinsic parameter matrix K describes the mapping relationship from the camera coordinate system to the image pixel coordinate system, and its form is as follows:

(11) $$K=\left[\begin{array}{ccc} f_{x} & 0 & c_{x}\\ 0 & f_{y} & c_{y}\\ 0 & 0 & 1 \end{array}\right]$$

Among them, $f_{x}$ and $f_{y}$ represent the focal lengths of the camera in the *x* and *y* directions, respectively, and $\left(c_{x},c_{y}\right)$ are the principal point coordinates of the image. Then, the point $\left(X_{c},Y_{c},Z_{c}\right)$ in the camera coordinate system corresponding to any pixel point (u,v) in the image coordinate system can be derived according to Formula (12):

(12) $$\left[\begin{array}{c} X_{c}\\ Y_{c}\\ Z_{c} \end{array}\right]=Z_{c}\cdot K^{-1}\left[\begin{array}{c} u\\ v\\ 1 \end{array}\right]$$

Meanwhile, the pixel point (u,v) in the image pixel coordinate system corresponding to the point $\left(X_{c},Y_{c},Z_{c}\right)$ in the camera coordinate system can be derived according to Formula (13):

(13) $$\left[\begin{array}{c} u\\ v\\ 1 \end{array}\right]=K\cdot \left[\begin{array}{c} \frac{X_{c}}{Z_{c}}\\ \frac{Y_{c}}{Z_{c}}\\ 1 \end{array}\right]$$

Thus, the mapping relationship between pixel points and 3D points is established, enabling bidirectional access between the image pixel coordinate system and the camera coordinate system.

### 3.5. Spatial Localization Combined with Depth Information

In the task of spatial localization in 3D scenes, the effective utilization of depth information is crucial for achieving high-precision localization [23]. This method completes the accurate mapping from RGB features to 3D coordinates through three core steps: depth image segmentation, 3D surface fitting, and spatial key point calculation. The specific process is as follows:

- Depth Image Segmentation

**Figure 6:**
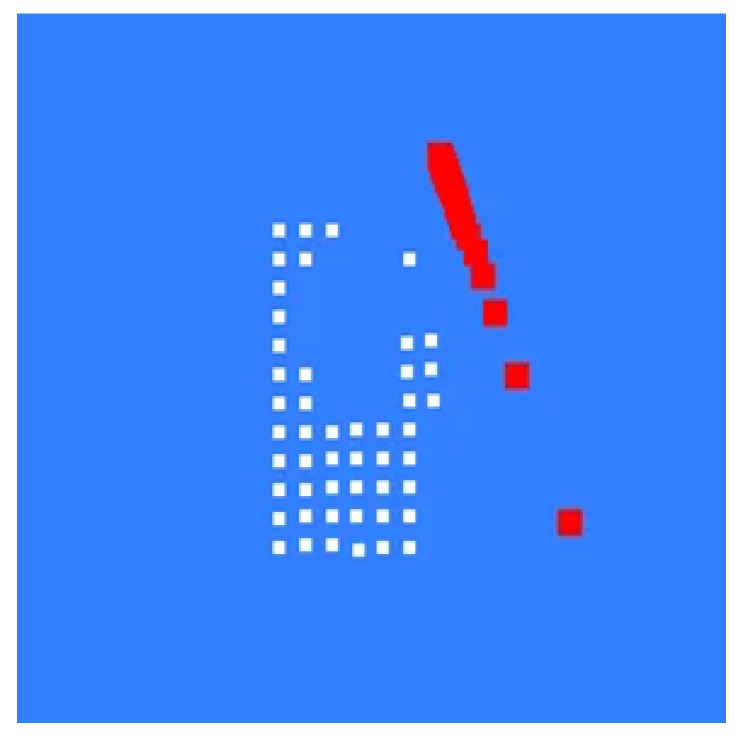
Extraction of the Target Region.

Depth images contain distance information between various objects in the scene and the depth camera, and depth edges usually correspond to geometric mutations on object surfaces or boundaries between different objects. Based on this characteristic, this paper first performs target region segmentation on the RGB image to clarify the range of the target workpiece in the RGB image. Subsequently, using the mapping relationship between RGB pixels and point cloud points, the boundary of the target region segmented from the RGB image is mapped to the depth image and the corresponding point cloud space, thereby separating the region belonging to the target workpiece in the point cloud, as shown in Figure 6. This provides targeted data foundation for subsequent surface fitting and effectively eliminates interference from the background and non-target regions.

- Quadratic Surface Fitting

For the subset of the workpiece region obtained by segmentation, although the corresponding 3D point cloud data exhibits characteristics of a continuous spatial surface, it may contain outliers due to factors such as measurement noise and equipment errors, which affect the accuracy of surface fitting. Therefore, it is necessary to preprocess the point cloud of the target region through a gross error detection mechanism before fitting: a statistics-based outlier detection method is adopted. First, the mean and standard deviation of the point cloud data are calculated, and points deviating from the mean by more than 3 times the standard deviation are identified as outliers. Discrete points that do not conform to the overall distribution trend of the surface are eliminated to ensure the reliability of the point cloud data used for fitting.

**Figure 7:**
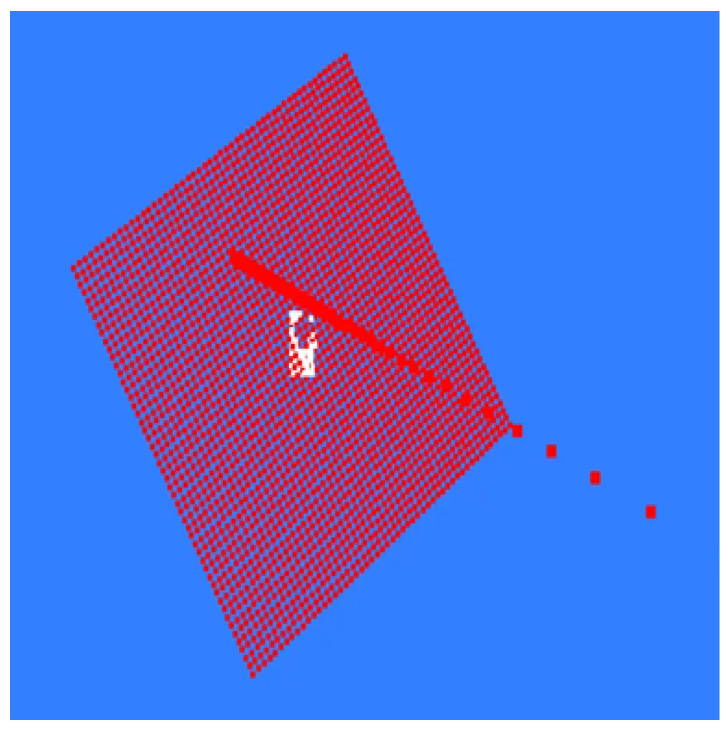
Quadratic Surface Fitting of the Target Region.

After preprocessing, to accurately describe the geometric shape of the surface, a spatial quadratic polynomial is used to fit the cleaned point cloud data. Quadratic polynomials can effectively characterize common geometric structures in industrial scenarios, such as planes and circular arc surfaces. While ensuring fitting accuracy, they also exhibit excellent numerical stability and computational efficiency, avoiding the overfitting risk associated with higher-order polynomials. After fitting, a surface equation in the form of z=f(x,y) is obtained (where *x* and *y* are the planar coordinates in the depth camera coordinate system, and *z* is the corresponding depth value). This equation fully characterizes the morphological features of the workpiece surface in 3D space, as shown in Figure 7.

- Calculation of Spatial Key Points

The localization of spatial key points requires the fusion of information from RGB images and depth images. First, in the RGB camera coordinate system, a spatial line $L_{0}$ is constructed by connecting the key points extracted from the RGB image with the physical optical center (origin) of the RGB camera as the starting point. This line represents the spatial direction from the camera’s perspective to the key point. Subsequently, using the extrinsic parameters between the RGB camera and the depth camera (i.e., the rotation matrix and translation vector describing the relative position and attitude relationship between the two cameras), the line $L_{0}$ is subjected to coordinate transformation and converted to the depth camera coordinate system, resulting in the corresponding spatial line $L_{1}$, which realizes the unification of spatial directions in different coordinate systems. Finally, the intersection point between the line $L_{1}$ and the previously fitted surface equation z=f(x,y) is solved. This intersection point is the 3D coordinate corresponding to the key point in the RGB image under the depth camera coordinate system, thus completing the accurate conversion from 2D image features to 3D spatial coordinates.

Through the above steps, spatial localization based on depth information is realized. It not only utilizes the 3D geometric constraints of depth images but also combines the feature recognition capability of RGB images, providing reliable 3D coordinate information of key points for subsequent applications such as 3D measurement and object pose estimation.

## 4. Results and Discussion

### 4.1. Test Data and Hardware Configuration

**Figure 8:**
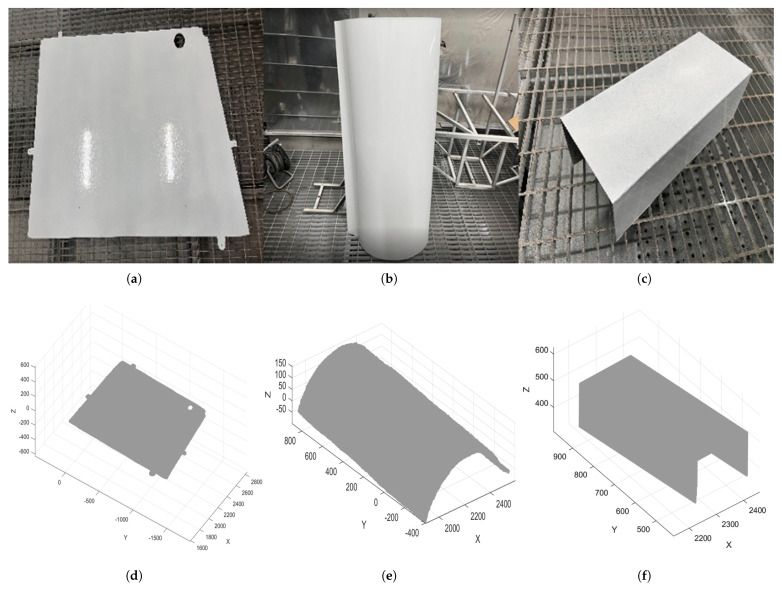
Schematic Diagrams of Three Typical Component Types. (**a**) Physical flat plate component; (**b**) Physical free-form surface component; (**c**) Physical groove component; (**d**) 3D model of free-form surface component; (**e**) 3D model of flat plate component; (**f**) 3D model of groove component.

To verify the feasibility and performance of the method proposed in this paper, a target component set and its 3D model feature database were established independently. All physical components involved were provided by AVIC Harbin Aircraft Industry Group Co., Ltd. (Harbin, China). For generality, three typical components and their 3D models, as shown in Figure 8, were selected from the target set as experimental objects for target positioning experiments. These components include flat plate type, free-form surface type, and groove type, covering most shapes of common components.

**Figure 9:**
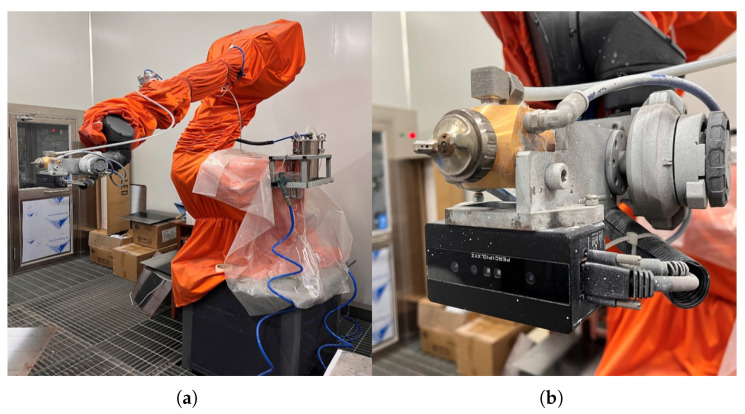
Schematic Diagram of Experimental Equipment. (**a**) Six-axis industrial robot; (**b**) Binocular camera.

In terms of hardware, this paper adopts the point cloud acquisition system shown in Figure 9a as the experimental platform. The system consists of an EFORT GR680 six-axis robot, a Tuyang FS-820 binocular camera, and a PC host. The camera is shown in Figure 9b, and the hardware parameters of the PC host are listed in Table 4 below. In terms of software, Python 3.11 is used for the training of the deep learning network model. Subsequently, the model is deployed on the C++ side via TorchScript scripts, and the algorithm and interface functions are implemented based on the MFC framework.

### 4.2. Performance Evaluation Metrics for the Method

This chapter selects the following evaluation metrics to comprehensively assess the performance of the target localization method proposed in this paper.

(1) Detection Reliability Metric

• Precision: Precision refers to the proportion of actually true samples among all samples detected by the model, as shown in Formula (14), where *TP* (True Positive) is the number of correctly detected positive samples, and *FP* (False Positive) is the number of falsely detected positive samples.

• Recall: Recall refers to the proportion of actually positive samples that are correctly predicted as positive, as shown in Formula (15), where *FN* (False Negative) is the number of missed positive samples.

• mAP@0.5: Mean Average Precision is the average of the average precisions of multiple categories, as shown in Formula (16). Here, *N* represents the number of target categories, and $AP_{i}$ represents the average precision of the i-th category.

(14) $$\begin{array}{cc} \mathrm{Precision} & =\frac{TP}{TP+FP} \end{array}$$

(15) $$\begin{array}{cc} \mathrm{Recall} & =\frac{TP}{TP+FN} \end{array}$$

(16) $$\begin{array}{cc} \mathrm{mAP} & =\frac{1}{N}\sum_{i=1}^{N}AP_{i} \end{array}$$

(2) Localization Accuracy Metric

• MAE: Mean Absolute Error refers to the average of the absolute values of Euclidean distances between the points in the 3D model after positioning and their corresponding points in the actual target object. It intuitively reflects the overall error level between the 3D model (post-positioning) and the actual target object, and does not fluctuate significantly due to individual outliers, as shown in Formula (17). Here, $p_{i}$ represents the coordinates of the i-th point in the 3D model after positioning, $q_{i}$ represents the coordinates of the corresponding point in the actual object, and n represents the number of points involved in the evaluation.

• RMSE: Root Mean Squared Error is calculated by first squaring the differences between the points in the 3D model after positioning and their corresponding points in the actual target object, then taking the average of these squared differences, and finally computing the square root of the average. Compared with Mean Absolute Error (MAE), this metric is more sensitive to large errors, as shown in Formula (18). Here, $p_{i}$ denotes the coordinates of the i-th point in the 3D model after positioning, $q_{i}$ denotes the coordinates of the corresponding point in the actual object, and n denotes the number of feature points involved in the evaluation.

• Max Error: The maximum error is the largest value among the localization errors of all feature points, reflecting the worst-case scenario of localization performance, as shown in Formula (19), where $\left(x_{i}^{\mathrm{pred}},y_{i}^{\mathrm{pred}},z_{i}^{\mathrm{pred}}\right)$ are the predicted coordinates and $\left(x_{i}^{\mathrm{true}},y_{i}^{\mathrm{true}},z_{i}^{\mathrm{true}}\right)$ are the ground truth coordinates.

• Standard Deviation: The standard deviation reflects the dispersion of localization errors and indicates the repeatability stability of the method. The calculation formula is shown in Formula (20), where $e_{i}$ represents the localization error of a single feature point, and $\bar{e}$ denotes the mean error. A smaller standard deviation indicates more stable repeat localization performance.

(17) $$\begin{array}{cc} \mathrm{MAE} & =\frac{1}{n}\sum_{i=1}^{n}\left|q_{i}-p_{i}\right| \end{array}$$

(18) $$\begin{array}{cc} \mathrm{RMSE} & =\sqrt{\frac{1}{n}\sum_{i=1}^{n}{\left(q_{i}-p_{i}\right)}^{2}} \end{array}$$

(19) $$\begin{array}{cc} \mathrm{Max}\;\mathrm{Error} & =\max\sqrt{{\left(x_{i}^{\mathrm{pred}}-x_{i}^{\mathrm{true}}\right)}^{2}+{\left(y_{i}^{\mathrm{pred}}-y_{i}^{\mathrm{true}}\right)}^{2}+{\left(z_{i}^{\mathrm{pred}}-z_{i}^{\mathrm{true}}\right)}^{2}} \end{array}$$

(20) $$\begin{array}{cc} \sigma & =\sqrt{\frac{1}{n-1}\sum_{i=1}^{n}{\left(e_{i}-\bar{e}\right)}^{2}} \end{array}$$

(3) Processing Efficiency Metric

• Average Processing Time: The average time required to process a single image frame (Unit: millisecond, ms), used to measure the core speed of the algorithm.

• FPS (Frames Per Second): The number of image frames that the system can process per second. It is the reciprocal of the average processing time (FPS=1000/AverageProcessingTime(ms)) and serves as an intuitive reflection of the real-time performance of industrial vision systems.

• Temporal Stability: The fluctuation of processing time during long-term continuous operation. It is usually represented by the standard deviation or range of processing time, reflecting the robustness of system performance.

(4) Edge Extraction Metric

• Edge Matching Rate (EMR): Measures the overlap between extracted edges and ground truth edges, reflecting the completeness of edge extraction, as shown in Formula (21), where $N_{\mathrm{overlap}}$ is the number of overlapping pixels between extracted edges and ground truth edges, and $N_{\mathrm{ground}\;\mathrm{truth}}$ is the total number of pixels in the ground truth edges. A higher EMR indicates more complete edge extraction.

• Edge Localization Error (ELE): Measures the pixel-level offset between extracted edges and ground truth edges, reflecting the precision of edge localization, as shown in Formula (22), where $\left(x_{i},y_{i}\right)$ are the coordinates of extracted edge pixels, $\left(x_{i}^{*},y_{i}^{*}\right)$ are the coordinates of corresponding ground truth edge pixels, and $N_{\mathrm{detected}}$ is the number of extracted edge pixels. The unit of ELE is “pixels”, and a smaller value indicates more precise edge localization.

• Noise Robustness Coefficient (NRC): The ratio of edge localization error with noise to that without noise, reflecting the noise resistance capability, as shown in Formula (23), where ${\mathrm{ELE}}_{\mathrm{noise}}$ is the edge localization error with added noise, and ${\mathrm{ELE}}_{\mathrm{clean}}$ is the edge localization error without noise. An NRC closer to 1 indicates that the operator is less affected by noise. Additionally, the standard deviation of NRC under different noise intensities is calculated, where a smaller standard deviation indicates stronger stability in noise resistance.

(21) $$\begin{array}{cc} \mathrm{EMR} & =\frac{N_{\mathrm{overlap}}}{N_{\mathrm{ground}\;\mathrm{truth}}}\times 100\% \end{array}$$

(22) $$\begin{array}{cc} \mathrm{ELE} & =\frac{1}{N_{\mathrm{detected}}}\sum_{i=1}^{N_{\mathrm{detected}}}\sqrt{{\left(x_{i}-x_{i}^{*}\right)}^{2}+{\left(y_{i}-y_{i}^{*}\right)}^{2}} \end{array}$$

(23) $$\begin{array}{cc} \mathrm{NRC} & =\frac{{\mathrm{ELE}}_{\mathrm{noise}}}{{\mathrm{ELE}}_{\mathrm{clean}}} \end{array}$$

### 4.3. Comparison of Network Model Performance and Ablation Experiments on the Overall Framework

(1) Comparison of Baseline Detection Models

To verify the effectiveness of different target detection models in the field of component feature detection studied in this paper, three network models from the YOLO series—YOLOv5, YOLOv8, and YOLOv10—are selected for experimental comparison on the self-built dataset with the same parameter settings. The experimental results are shown in Table 5.

Based on the data analysis in Table 5, YOLOv10 achieves the highest detection precision and mean average precision (MAP) with a fast inference speed, thus being selected as the local component feature detection model in this paper. We note that the field of object detection is evolving rapidly, with new variants continuously proposed. This comparative experiment aims to select a backbone detector with excellent comprehensive performance for our framework from the representative versions of the YOLO series, and the experimental results support the choice of YOLOv10. The core advantage of the proposed method lies in its backend fusion and localization pipeline, which maintains openness and compatibility with frontend detection modules.

(2) Ablation Experiments on the Overall Framework

• A. Background Misdetection Filtering Module (BMFM): Enhances the robustness of coarse localization.

• B. Improved Prewitt Operator and Sub-pixel Key Point Fitting: Improves the localization accuracy of 2D feature points.

• C. Fine Localization via Ray Intersection Based on Quadratic Surface Fitting: Replaces the simple point cloud nearest neighbor mapping to enhance 3D localization accuracy.

We designed the following ablation experimental scheme: taking “YOLOv10 coarse localization + traditional edge extraction (Canny) + point cloud nearest neighbor mapping” as the baseline method, we gradually added our proposed components and evaluated the final 3D localization accuracy (MAE) and processing efficiency on the test set. The results are shown in Table 6.

Role of the BMFM Module: By comparing Experiments 1 and 2, it is observed that after introducing the Background Mis-detection Filtering Module (BMFM), the Mean Absolute Error (MAE) decreases from 0.42 mm to 0.40 mm (a reduction of 4.8%), and the Root Mean Squared Error (RMSE) decreases from 0.49 mm to 0.46 mm. Meanwhile, the recall rate remains nearly unchanged (0.830 → 0.834). This indicates that the BMFM module can effectively filter out background mis-detected regions (reducing False Positives, FP), avoiding interference from background point clouds in subsequent localization. Additionally, it only increases the processing time by 0.8 ms, demonstrating excellent efficiency.

Role of Improved Edge Extraction and Sub-pixel Localization: By comparing Experiments 1 and 3, after replacing traditional Canny edge extraction with the improved Prewitt operator combined with sub-pixel fitting, the MAE decreases significantly by 31.0% (0.42 → 0.29 mm) and the RMSE decreases by 32.7% (0.49 → 0.33 mm). This is attributed to the improved operator’s enhanced edge matching rate and localization accuracy, which provides a more reliable 2D foundation for the calculation of sub-pixel key points.

Role of Surface Fitting-based Precise Localization: By comparing Experiments 1 and 4, after replacing simple point cloud nearest-neighbor mapping with surface fitting and ray intersection, the MAE decreases by 40.5% (0.42 → 0.25 mm) and the RMSE decreases by 38.8% (0.49 → 0.30 mm). This result validates the core argument: when point clouds are sparse or missing at key feature points, reconstructing the geometric shape through surface fitting is more accurate and robust than directly using raw point cloud data.

Synergistic Effect of Components: In Experiment 5 (the complete method), after integrating all components, the MAE is further reduced to 0.17 mm and the RMSE to 0.18 mm. Moreover, the average processing time per frame is 17.2 ms (≈58.1 FPS), which meets the real-time requirements of industrial scenarios. This indicates that the components do not function as a simple superposition but rather exhibit a synergistic effect: the BMFM provides clean detection regions, the improved operator delivers precise 2D edges, and surface fitting enables 3D geometric reconstruction—collectively supporting sub-millimeter-level localization accuracy.

(3) Ablation Experiment on Edge Extraction Operators: To verify the superiority of the improved Prewitt operator (8-directional 5 × 5 convolution kernel) proposed in this paper in terms of edge extraction accuracy and anti-noise capability, a dedicated comparative experiment was conducted on the self-built dataset using traditional edge extraction operators as the control group. For fairness of comparison, all other processes (sub-pixel fitting, 3D mapping, and surface fitting) remained unchanged throughout the experiments, with only the edge extraction module replaced. Control Group: Traditional Prewitt operator (2-directional 3 × 3 convolution kernel); Sobel operator (2-directional 3 × 3 convolution kernel, with weights [1,2,1] for the x-direction and [1,0,−1] for the y-direction); Canny operator (double thresholds set to 50/150, with a 3 × 3 Gaussian filter). Experimental Group: The improved Prewitt operator proposed in this paper (8-directional 5 × 5 convolution kernel, covering directions of 0,π/4,π/2,3π/4,π,5π/4,6π/4,7π/4). For the experiment, 50 noise-free images were selected from each of the flat plate, free-form surface, and groove component datasets, along with images with added noise (Gaussian noise with σ = 0.05/0.1/0.15, salt-and-pepper noise with density = 0.01/0.03/0.05). The ground-truth edges were generated by projecting high-precision 3D models (with an error ≤0.02 mm) onto 2D images.

• Advantage in Edge Extraction Accuracy: As shown in Table 7, in the noise-free scenario, the EMR (Edge Matching Rate) of the proposed improved Prewitt operator reaches 91.8%, which is 10.3%, 7.6%, and 4.5% higher than that of the traditional Prewitt (81.5%), Sobel (84.2%), and Canny (87.3%) operators, respectively. Its ELE (Edge Localization Error) is only 0.45 pixels, representing a 35.7% reduction compared to the Canny operator (0.70 pixels). This superiority is attributed to the complete coverage of diagonal edges by the 8-directional convolution kernel and the full utilization of local edge information by the 5 × 5 kernel, which avoids the missed detection of diagonal features common in traditional 2-directional operators.

• Advantage in Anti-Noise Capability: After adding Gaussian noise with σ = 0.1, the ELE of the improved operator increases to 0.71 pixels, with an NRC (Noise Robustness Coefficient) of 1.58 (the closest to 1). Moreover, the standard deviation of NRC across different noise intensities is merely 0.11, significantly lower than that of the Canny operator (0.22) and traditional Prewitt operator (0.18). The smoothing effect of the 5 × 5 convolution kernel effectively suppresses noise interference, while the 8-directional feature fusion further reduces edge deviation caused by noise.

• Balance between Efficiency and Performance: The edge extraction time of the improved operator is 2.3 ms. Although this is longer than that of traditional 3 × 3 operators (0.9–1.5 ms), it only accounts for 13.4% of the total processing time of the entire positioning workflow (17.2 ms). Furthermore, the improved accuracy directly contributes to a 32.3% reduction in the subsequent sub-pixel positioning error.

(4) Validation of the BMFM Module’s Effectiveness

**Figure 10:**
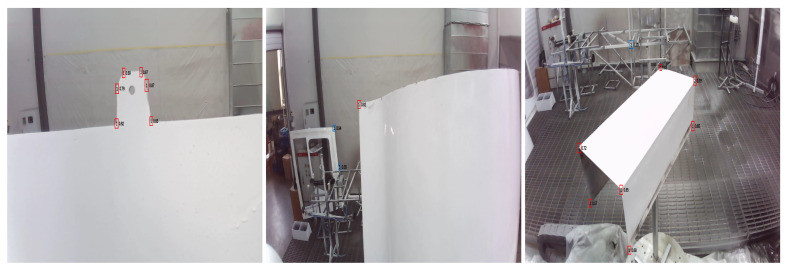
Schematic diagram of misdetected feature filtering for some components.

To independently verify the performance of the BMFM module in the object detection phase, we conducted ablation experiments on the component dataset. Partial results of false detection feature filtering are illustrated in Figure 10, where blue bounding boxes represent the filtered background false detection regions and red bounding boxes denote the retained component feature regions.

As shown in Table 8, the BMFM module increases the detection precision by 9.58% while maintaining the original high recall rate. The F1-Score rises from 0.833 to 0.872, and the mean Average Precision (mAP@0.5) also increases from 0.879 to 0.902. This indicates that the module can accurately identify and filter out false detections caused by complex backgrounds without affecting the detection of real features. The additional time cost is only 0.8 ms (a 21% increase), which is within an acceptable range, providing cleaner and more reliable input for subsequent processes.

(5) System Real-Time Performance and Comparative Analysis

To comprehensively evaluate the overall performance of the proposed method in industrial real-time application scenarios, we conducted a detailed time-consuming analysis of the complete workflow (i.e., the “Proposed Complete Method” in Table 6). The time consumption of each phase is presented in Table 9.

The results indicate that the average total processing time of the proposed method for a single frame is 17.2 ms, corresponding to a system throughput of approximately 58.1 FPS (frames per second). To verify the system’s stability during continuous operation, tests were conducted on a sequence of 1000 consecutive frames. The processing time fluctuates between 15.8 ms and 18.9 ms with a standard deviation of 0.71 ms, demonstrating excellent temporal stability. This performance meets the real-time processing requirement of ≥30 FPS typically demanded by industrial vision systems.

While recent industrial studies have made significant progress in high-speed detection, such as the lightweight KPD model reported by Lu et al. [24] that achieves remarkable real-time performance on embedded devices, the proposed method adopts a different optimization strategy. Rather than pursuing maximum processing speed, our approach strategically balances computational efficiency with positioning accuracy. This design philosophy enables the maintenance of ultra-high submillimeter-level positioning accuracy while providing satisfactory real-time processing capability. Such a balanced performance profile makes the method particularly suitable for precision-critical industrial applications where both accuracy and timeliness are essential, including precision manufacturing, aerospace component inspection, and other high-stakes quality assurance scenarios.

### 4.4. Experiment for Verifying the Accurate Localization Precision of the Target Model

**Figure 11:**
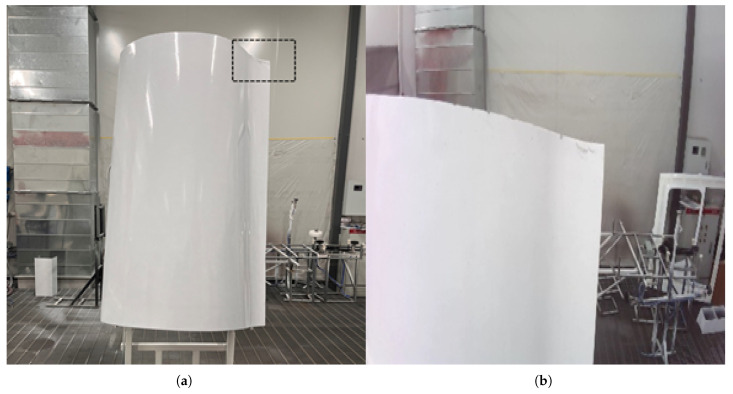
Schematic Diagram of Initial Viewpoint Acquisition: (**a**) Free-form Surface Components; (**b**) RGB Image at Viewpoint $V_{1}$.

To verify the accuracy of the proposed method in accurately localizing the target model, the experimental process and results are as follows: First, the free-form surface component shown in Figure 11a is installed on the system positioner, and an initial viewpoint $V_{1}$ is given so that the camera can collect the RGB image $I_{1}$ near the feature points and the local point cloud $P_{1}$. $I_{1}$ and $P_{1}$ are shown in Figure 11b, respectively.

By processing the RGB image $I_{1}$ using the method in Section 3.3, the feature region $F_{1}$ (as shown in Figure 11a) and the sub-pixel intersection coordinates $C_{1}^{2}\left(1122.31,420.58\right)$ of the two edges in the image (as shown in Figure 11b) can be obtained.

For this sub-pixel point, the specific data and steps for ray generation and intersection calculation, in conjunction with Section 3.5, are as follows:

1. Ray Generation: Based on the calibrated intrinsic parameter matrix {K} of the color camera

$$\left[\begin{array}{ccc} 1215.3 & 0 & 640.2\\ 0 & 1218.7 & 360.5\\ 0 & 0 & 1 \end{array}\right]$$

Ray Generation: The sub-pixel point $C_{1}^{2}\left(1122.31,420.58\right)$ is projected into the color camera coordinate system to generate a spatial ray. This ray starts from the camera optical center $O_{c}\left(0,0,0\right)$ and has a direction vector d=(0.821,0.345,1.000). Its parametric equation is expressed as:

r(t)=(0,0,0)+t·(0.821,0.345,1.000),t≥0

2. Coordinate System Transformation: Using the external parameters between the color camera and the depth camera (rotation matrix R and translation vector t):

$$R=\left[\begin{array}{ccc} 0.999 & -0.012 & 0.035\\ 0.011 & 0.999 & -0.018\\ -0.035 & 0.017 & 0.999 \end{array}\right],\;t=\left(5.2,-3.1,10.8\right)$$

The ray r(t) is transformed into the depth camera coordinate system (consistent with the point cloud coordinate system), resulting in the transformed ray equation:

$$R^{\prime }\left(t\right)=\left(5.2,-3.1,10.8\right)+t\cdot \left(0.819,0.352,0.998\right),\;t\geq 0$$

3. Surface Intersection Calculation: The point cloud of the target region obtained by depth image segmentation is fitted with a quadratic polynomial surface, and the resulting surface equation is:

$$z=0.0002x^{2}+0.0001y^{2}-0.003xy+0.05x-0.02y+1200.5$$

By solving the intersection of the ray $R^{\prime }\left(t\right)$ and this fitted surface, the precise 3D spatial coordinates of the feature point are finally calculated: $C_{1}^{3}\left(2410.05,-292.78,1294.24\right)$

**Figure 12:**
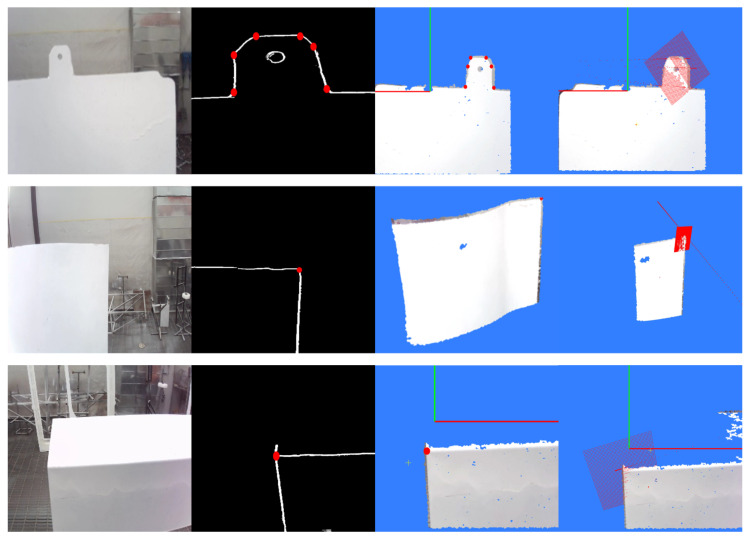
Schematic Diagram of Feature Point Extraction at Viewpoint $V_{1}$: Region of Feature Points, Image Feature Point Extraction, Point Cloud Feature Point Extraction, and Accurate Feature Point Extraction.

This process clearly demonstrates the complete technical chain from 2D image feature detection to 3D spatial ray generation, and finally to precise positioning through geometric intersection calculation, as shown in Figure 12. This example provides a foundation for subsequent statistical accuracy verification with large samples.

To achieve submillimeter-level accuracy verification, a reliable reference benchmark is required. In practical industrial inspection and on-line verification scenarios, factors such as cost constraints, limited workspace, and the demand for rapid deployment often make it impractical to frequently use high-end stationary metrology equipment (e.g., coordinate measuring machines or laser trackers). This study employs a vision-based measurement scheme that utilizes a high-precision steel ruler conforming to the Chinese National Standard GB/T 9056-2004 “Metal Straight Rule” [25] as a traceable physical reference. The standard specifies a scale interval of 1 mm. For a ruler of length L (in mm), the maximum permissible error is ±(0.10 + 0.05 × L/500) mm. The ruler used in the experiments has a calculated inherent accuracy better than ±0.15 mm, which, from the perspective of national standardization, ensures its reliability as a length datum and matches the sub-millimeter accuracy level of the localization results to be validated.

The core objective of this verification scheme is not to use instantaneous ruler readings as absolute ground truth, but to obtain a highly stable and reproducible spatial coordinate benchmark through a rigorous multi-view vision measurement and data fusion process. The reliability of this benchmark is guaranteed at two levels:

First Level: Traceable Physical Foundation. The steel ruler, as a standardized material measure, provides stable and known scale intervals. It is rigidly connected to the workpiece, becoming a fixed part of the measurement coordinate system with known accuracy, thereby establishing a traceable physical foundation for subsequent visual measurements.

Second Level: High-Repeatability Measurement Process and Stability Verification. Utilizing the sub-pixel edge detection technique proposed in this paper (Section 3.2 and Section 3.3), the ruler scales and workpiece feature points are simultaneously imaged and localized from multiple (e.g., six) viewpoints. Through camera calibration parameters and multi-view triangulation, the 3D coordinates of the feature points are calculated independently for each viewpoint, yielding multiple sets of “single-viewpoint observations.” The crucial step is stability verification: a final benchmark coordinate is obtained through weighted fusion of these observations. To confirm the reliability of this benchmark, a repeatability test was conducted: the entire process from image acquisition to data fusion was independently executed four times. The maximum deviation among the benchmark coordinates obtained from these four independent repetitions was less than 0.04 mm for all feature points.

In summary, this scheme ultimately provides a set of “spatial coordinate benchmarks verified by multi-view measurement and exhibiting high repeatability.” This benchmark is rooted in a standardized physical entity, and its stability is demonstrated through a rigorous process. Its uncertainty (<0.04 mm) is significantly smaller than the localization error of the proposed method (0.17 mm), and it is therefore entirely sufficient to serve as a reliable basis for evaluating the localization accuracy of the proposed algorithm.

The specific scheme to implement this process is as follows:

(1) Fixation and Collaborative Calibration of Steel Ruler and Workpiece

A high-precision steel ruler conforming to GB/T 9056-2004 (scale interval: 1.0 mm) is rigidly fixed to the workpiece using a custom-made mechanical fixture. This establishes a unified and traceable reference coordinate system between the ruler and the workpiece. Rigid connections are ensured to avoid relative displacement during the measurement process.

(2) Design of Multi-Angle Measurement Scheme

To ensure complete and high-precision observation, 6 uniformly distributed measurement viewpoints are positioned within the upper hemisphere of the plane where the feature points are located. The optical axes of all cameras are directed toward a shared measurement area. Each viewpoint must meet the following conditions:

• Both the scale of the steel ruler and the workpiece’s feature points are within the clear field of view, with no occlusion;

• The included angle between the camera’s optical axis and the plane of the feature points ranges from 30° to 60°;

• The imaging overlap rate of the steel ruler and feature points between adjacent viewpoints is not less than 70%.

All viewpoints are equipped with uniformly calibrated RGB-D cameras of the same model, establishing a global measurement coordinate system.

(3) Benchmark Acquisition via Multi-View Measurement and Fusion

• Image Acquisition and Coordinate Calculation: At each viewpoint, images are captured. The sub-pixel edge detection technique (Section 3.2 and Section 3.3) is employed to locate the ruler’s scale marks and workpiece feature points. 3D coordinates are then calculated independently for each viewpoint via camera calibration and triangulation, yielding six sets of “single-viewpoint observations.”

• Data Fusion: Based on the global measurement coordinate system, weighted fusion is performed on the “single-viewpoint observations” from the 6 viewpoints. The weight is determined by the imaging clarity (e.g., grayscale contrast) of the steel ruler’s scale at each viewpoint. Through optimization using the least squares method, the final 3D benchmark coordinates of the feature points are obtained.

(4) Verification of Benchmark Stability and Reliability

• Repeatability of the Entire Process: The complete multi-view measurement and fusion process (Steps 1–3) was independently repeated four times. The maximum deviation among the final benchmark coordinates obtained from these four independent repetitions was less than 0.04 mm for all feature points. This conclusively proves the high repeatability and stability of the benchmark acquisition process.

• Calibration of the Physical Reference: Standard gauge blocks were used to calibrate the steel ruler’s key scales, ensuring its scale error was less than 0.01 mm, thus eliminating tool error as a significant influence on the benchmark.

For each feature point localized by the proposed method, the Euclidean distance between its predicted 3D coordinates and the corresponding benchmark coordinate obtained above is calculated as the localization error using Formula (24).

(24) $$e_{i}=\sqrt{{\left(x_{i}^{\mathrm{pred}}-x_{i}^{\mathrm{true}}\right)}^{2}+{\left(y_{i}^{\mathrm{pred}}-y_{i}^{\mathrm{true}}\right)}^{2}+{\left(z_{i}^{\mathrm{pred}}-z_{i}^{\mathrm{true}}\right)}^{2}}$$

Among them, $\left(x_{i}^{\mathrm{pred}},y_{i}^{\mathrm{pred}},z_{i}^{\mathrm{pred}}\right)$ represents the coordinates of the feature points output by the method proposed in this paper, and $\left(x_{i}^{\mathrm{benchmark}},y_{i}^{\mathrm{benchmark}},z_{i}^{\mathrm{benchmark}}\right)$ denotes the benchmark coordinates obtained through the multi-view measurement and fusion process described in Section 4.4.

To rigorously verify the positioning accuracy and stability of the proposed method, we tested 15 feature points for each component type (Flat Plate, Free-form Surface, and Groove), resulting in a total of 45 test instances. The positioning error of each feature point (defined as the Euclidean distance between its predicted coordinates and the corresponding benchmark coordinates) was recorded and presented in Table 10. This table also provides statistical metrics calculated based on all samples, including Mean Absolute Error (MAE), Root Mean Square Error (RMSE), Maximum Error, and Standard Deviation.

Statistical analysis results indicate that the proposed method achieves stable submillimeter-level positioning accuracy for the three types of typical components. As shown in Table 11, the overall average Mean Absolute Error (MAE) across the three components is 0.17 mm; the overall average Root Mean Square Error (RMSE) is 0.18 mm; and the maximum error is less than 0.22 mm for all cases. In addition, the small standard deviations (0.018–0.022 mm) demonstrate the method’s high repeat positioning accuracy and stable performance.

The analysis of error differences is as follows: Flat Plates exhibit the smallest error (average MAE = 0.15 mm) due to their flat surfaces, clear edges, and high stability in feature extraction. Moreover, the features can be clearly captured from all viewpoints during multi-angle measurements, resulting in higher benchmark accuracy. Free-form Surfaces show slightly larger errors (average MAE = 0.17 mm). Affected by the curvature variation of the surface, the point cloud density around feature points is relatively low from some viewpoints, leading to minor deviations in benchmark calculation. Meanwhile, the increased difficulty of surface fitting also introduces additional errors. Grooves present the largest errors (average MAE = 0.18 mm). Their groove structure is prone to view occlusion, making it impossible to fully collect feature point information from certain viewpoints. Although compensation is achieved through overlapping fields of view, the benchmark accuracy is still reduced. Additionally, the high complexity of groove edges affects the accuracy of feature extraction.

The MAE and RMSE of all experimental results are significantly lower than those of traditional methods. Combined with detailed individual error data and high-precision benchmark-based verification, these results fully demonstrate the high accuracy and reliability of the proposed method. They also validate the effectiveness of the “steel ruler + multi-angle repeated measurement” scheme in scenarios where professional measurement equipment is unavailable.

### 4.5. Comparative Experiments

To verify the effectiveness and advancement of the proposed method in this paper, typical free-form surface components and groove components in Figure 8 were selected as experimental objects, and the point cloud acquisition system shown in Figure 9 was uniformly used as the experimental platform. Three representative baseline methods were selected for comparative experiments in this paper, and a comprehensive evaluation was conducted from two dimensions: positioning accuracy and processing efficiency. Detailed comparison results for flat plate, free-form surface, and groove components are presented in Table 12, Table 13, and Table 14, respectively.

The processing time for our proposed method is reported as 17.2 ms for all component types, representing the average processing time across multiple trials and component types. This provides a consistent basis for comparison with other methods.

### 4.6. Chapter Summary

This chapter elaborates on the high-precision positioning method for curved surface feature points based on RGB-D data. The specific process includes the following steps: First, two types of feature regions—corner surface key points and spatial cusp key points—are defined, and a dataset for local component feature detection is established. To address the problem of background false detection in unstructured environments, a Background Misdetection Filtering Module (BMFM) is proposed, and the YOLOv10 model is used to complete the coarse positioning of feature regions.

Second, an improved Prewitt operator (extended to an 8-direction 5 × 5 convolution kernel) is applied to extract foreground contours from feature region images. Combined with binocular vision depth information to filter out background interference, continuous sub-edges are obtained through 8-direction gradient decomposition. Polynomial fitting is used for the sub-edges to solve the sub-pixel-level feature point coordinates (the intersection of two edges is solved directly by simultaneous equations, and the intersection of three edges is optimized through mean processing).

Furthermore, based on the binocular camera imaging principle, the feature boundaries extracted from 2D images are accurately mapped to the 3D point cloud space to determine the point cloud boundaries and complete the division of the target region. On this basis, preprocessing is performed on the target region point cloud obtained by depth image segmentation, and outliers are removed through a gross error detection mechanism to ensure data reliability. Then, a quadratic polynomial is used for 3D surface fitting. Finally, the spatial ray corresponding to the RGB feature point is intersected with the fitted surface to accurately locate the intersection between regions, obtain the 3D spatial key point coordinates, and realize high-precision spatial positioning of complex curved surface feature points.

Finally, experiments are conducted to verify the effectiveness of the method: Model performance comparison shows that YOLOv10 outperforms YOLOv5 and YOLOv8 in detection precision (0.835) and mean average precision (0.879), and the precision increases by 9.58% after adding the BMFM module. Positioning experiments on three typical component types (flat plate, free-form surface, and groove) indicate that the average MAE of the method is 0.17 mm, and the maximum error is less than 0.22 mm, achieving sub-pixel-level positioning accuracy and meeting the requirements of high-precision industrial applications.

## 5. Conclusions

This paper proposes a high-precision positioning method for feature points on complex curved surfaces based on RGB-D fusion, achieving accurate workpiece positioning through a three-level technical architecture of “feature classification–sub-pixel refinement–cross-modal fusion”: Firstly, two types of features—angular surface key points and spatial cusp key points—are clearly defined. Coarse positioning is completed based on the YOLOv10 model, combined with a Background Misdetection Filtering Module (BMFM) to enhance detection robustness; secondly, multi-directional edges are extracted using an improved Prewitt operator, and fine positioning of 2D feature points is realized through polynomial fitting and sub-pixel subdivision technology; finally, a cross-modal mapping mechanism between RGB and depth data is constructed, a 3D curved surface is fitted using a quadratic polynomial, and 3D positioning is completed by calculating the intersection of spatial rays with the fitted surface.

Experimental verification shows that the method achieves an average positioning error (MAE) of 0.17 mm and a maximum error of less than 0.22 mm for flat plate, free-form surface, and groove components, meeting the high-precision requirements of industrial inspection. Compared with traditional methods, the RGB-D fusion path adopted in this method stands in sharp contrast to stereo vision schemes that require multi-camera calibration and dense matching to recover depth. The proposed method fully leverages the characteristic of RGB-D sensors to synchronously provide texture and depth information. Through deep fusion of these two complementary data sources, it avoids problems such as matching failure in low-texture regions and easy drift of calibration parameters in stereo vision, while overcoming the difficulty of sparse data at feature points in pure point cloud methods. Ultimately, it significantly improves the positioning accuracy and operational robustness of feature points on complex curved surfaces.

Despite the aforementioned achievements, we recognize that the method has certain limitations. Firstly, the verification of the current research is focused on specific types of components and imaging conditions; the generalization performance of the method needs further systematic evaluation when facing significantly different material properties, extreme lighting changes, or a wider range of working distances. Secondly, the performance of the method largely depends on clear edge features of ridges in RGB images. For workpieces with smooth surfaces, lack of texture, or severe specular reflection, the stability of feature extraction will be affected. In addition, the current method targets feature points formed by the intersection of clear edges; its positioning capability for feature points with blurred features or more complex free-form surface morphologies still needs further verification. Finally, the real-time performance of the system is achieved under a specific hardware configuration, and whether the same performance can be maintained on embedded platforms with more limited computing resources remains to be explored.

Future research will focus on three aspects: Firstly, optimizing the computational bottlenecks in the algorithm process (such as the iterative efficiency of 3D curved surface fitting) to improve real-time performance in dynamic industrial scenarios, and exploring lightweight architectures to enhance applicability on embedded platforms; secondly, expanding the scope of application of the method, with a focus on studying how to improve positioning robustness and generalization ability under conditions such as smooth surfaces, lack of texture, complex specular reflection, and a wider range of industrial working conditions.

Future research will be carried out in three aspects:

• Optimization of process efficiency and deployment adaptability: Relying on the modular characteristics of the coarse localization module, newer versions such as YOLOv11 and YOLOv13 will be introduced (referring to the lightweight architecture of YOLOv13 proposed by Li et al. [26] in 2025 and the edge deployment research of YOLOv11 conducted by Diaz-Santos et al. [27] in 2025). On the premise of keeping the fine localization module (improved Prewitt operator + 3D surface fitting) unchanged, the false detection filtering pressure of the BMFM module will be reduced. Meanwhile, the iterative efficiency of 3D surface fitting will be optimized, and lightweight schemes such as model quantization will be explored to improve the deployment practicality on embedded platforms like Raspberry Pi and Jetson Nano, so as to adapt to dynamic scenarios such as high-speed production lines.

• Expansion of applicable working conditions: For complex working conditions including smooth surfaces, weak textures, and specular reflections, technologies such as polarization imaging and texture generation will be used to optimize the quality of RGB input. In combination with point cloud denoising and completion, the problem of sparse depth data will be improved. Additionally, a deep learning-driven prediction mechanism for surface feature points will be explored to break the reliance on “feature points formed by the intersection of clear edges” and enhance the localization ability for free-form surfaces.

• Enhancement of generalization performance evaluation: A diverse dataset covering multiple materials (such as metals and plastics), extreme lighting conditions, and different working distances will be constructed. By combining transfer learning and domain adaptation technologies, the impact of environmental differences will be reduced, thereby promoting the application of this method in fields such as aerospace and automobile manufacturing.

## Figures and Tables

**Table 1:** Table of Feature Region Categories.

Feature Region Type	Feature Region Description	Feature Region Example
Type 1	Intersection region of two edge lines	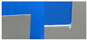
Type 2	Intersection region of three edge lines	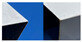

**Table 2:** Dataset Composition and Augmentation Details.

Component Type	Raw Images	Augmented Training Set	Validation Set	Test Set	Total
Flat Plate	439	2150	461	461	3072
Free-form Surface	513	2300	493	493	3286
Grooved	439	2150	461	461	3072
Total	1391	6600	1415	1415	9227

**Table 3:** Convolution Kernel Parameters of the Improved 8-Direction 5 × 5 Prewitt Operator.

Gradient Direction	5 × 5 Convolution Kernel
0	$\left[\begin{array}{ccccc} -2 & -1 & 0 & 1 & 2\\ -2 & -1 & 0 & 1 & 2\\ -2 & -1 & 0 & 1 & 2\\ -2 & -1 & 0 & 1 & 2\\ -2 & -1 & 0 & 1 & 2 \end{array}\right]$
π/4	$\left[\begin{array}{ccccc} -2 & -2 & -1 & 0 & 1\\ -2 & -1 & 0 & 1 & 2\\ -1 & 0 & 1 & 2 & 2\\ 0 & 1 & 2 & 2 & 2\\ 1 & 2 & 2 & 2 & 2 \end{array}\right]$
π/2	$\left[\begin{array}{ccccc} -2 & -2 & -2 & -2 & -2\\ -1 & -1 & -1 & -1 & -1\\ 0 & 0 & 0 & 0 & 0\\ 1 & 1 & 1 & 1 & 1\\ 2 & 2 & 2 & 2 & 2 \end{array}\right]$
3π/4	$\left[\begin{array}{ccccc} 1 & 0 & -1 & -2 & -2\\ 2 & 1 & 0 & -1 & -2\\ 2 & 2 & 1 & 0 & -1\\ 2 & 2 & 2 & 1 & 0\\ 2 & 2 & 2 & 2 & 1 \end{array}\right]$
π	$\left[\begin{array}{ccccc} 2 & 1 & 0 & -1 & -2\\ 2 & 1 & 0 & -1 & -2\\ 2 & 1 & 0 & -1 & -2\\ 2 & 1 & 0 & -1 & -2\\ 2 & 1 & 0 & -1 & -2 \end{array}\right]$
5π/4	$\left[\begin{array}{ccccc} 2 & 2 & 2 & 2 & 1\\ 2 & 2 & 2 & 1 & 0\\ 2 & 2 & 1 & 0 & -1\\ 2 & 1 & 0 & -1 & -2\\ 1 & 0 & -1 & -2 & -2 \end{array}\right]$
3π/2	$\left[\begin{array}{ccccc} 2 & 2 & 2 & 2 & 2\\ 1 & 1 & 1 & 1 & 1\\ 0 & 0 & 0 & 0 & 0\\ -1 & -1 & -1 & -1 & -1\\ -2 & -2 & -2 & -2 & -2 \end{array}\right]$
7π/4	$\left[\begin{array}{ccccc} 2 & 2 & 2 & 1 & 0\\ 2 & 2 & 1 & 0 & -1\\ 2 & 1 & 0 & -1 & -2\\ 1 & 0 & -1 & -2 & -2\\ 0 & -1 & -2 & -2 & -2 \end{array}\right]$

**Table 4:** Hardware Configuration Table of the Experimental Host.

Hardware Name	Hardware Configuration
Central Processing Unit (CPU)	Intel(R) Core(TM) i9-13900HX
Memory (RAM)	64 GB
Graphics Card (GPU)	NVIDIA GeForce RTX 4060 Laptop GPU
Graphics Memory	8 GB

**Table 5:** Performance Comparison of Different Object Detection Models.

Model Name	Precision	Recall	mAP@0.5	Inference Time
YOLOv5	0.812	0.832	0.874	4.7 ms
YOLOv8	0.833	0.773	0.847	3.5 ms
YOLOv10	0.835	0.830	0.879	3.8 ms

**Table 6:** Ablation Experiment Results of the Overall Framework.

Experiment ID	Method Configuration	MAE	RMSE	Total Processing Time per Frame (ms)
1	Baseline: YOLOv10 + Canny Edge Extraction + Point Cloud Nearest Neighbor Mapping	0.42	0.49	11.5
2	Baseline + A. BMFM Module	0.40	0.46	12.3
3	Baseline + B. Improved Edge and Sub-pixel Localization	0.29	0.33	14.7
4	Baseline + C. Surface Fitting-based Fine Localization	0.25	0.30	16.0
5	Proposed Complete Method: Baseline + A + B + C	0.17	0.18	17.2

**Table 7:** Comprehensive Comparison of Performance and Anti-Noise Capability of Different Edge Extraction Operators.

Operator Type	Noise-Free Scenario		σ = 0.1 Gaussian Noise Scenario			Time (ms)
	EMR (%)	ELE (Pixels)	ELE (Pixels)	NRC	NRC Std (Different Noise)	
Traditional Prewitt	81.5 ± 2.4	0.88 ± 0.09	1.45 ± 0.14	1.65	0.18	0.9
Sobel	84.2 ± 2.1	0.75 ± 0.08	1.32 ± 0.12	1.76	0.16	1.1
Canny	87.3 ± 1.9	0.70 ± 0.07	1.28 ± 0.11	1.83	0.22	1.5
Proposed Improved Prewitt	91.8 ± 1.4	0.45 ± 0.05	0.71 ± 0.07	1.58	0.11	2.3

Note: EMR = Edge Matching Rate; ELE = Edge Localization Error; NRC = Noise Robustness Coefficient; Std = Standard Deviation. All statistical results are presented as “mean ± standard deviation”.

**Table 8:** Results of Ablation Experiments on the BMFM Module.

Model Name	Precision	Recall	F1-Score	mAP@0.5	Feature Detection Time
YOLOv10	0.835	0.830	0.833	0.879	3.8 ms
YOLOv10 + BMFM	0.915	0.834	0.872	0.902	4.6 ms

**Table 9:** Average Processing Time of Each Stage.

Processing Stage	Average Time (ms)
YOLOv10 + BMFM Feature Region Detection	4.6
Improved Prewitt Edge Extraction and Decomposition	3.2
Sub-pixel Keypoint Fitting	1.5
Point Cloud Mapping and Target Region Extraction	2.8
Surface Fitting and Intersection Calculation	5.1
Total Processing Time per Frame	17.2

**Table 10:** Absolute Error $e_{i}$ of Different Component Types and Feature Points.

Flat Plate		Free-Form Surface		Groove	
Feature Point No.	Absolute Error $e_{i}$ (mm)	Feature Point No.	Absolute Error $e_{i}$ (mm)	Feature Point No.	Absolute Error $e_{i}$ (mm)
1	0.138	1	0.165	1	0.172
2	0.161	2	0.198	2	0.205
3	0.148	3	0.158	3	0.165
4	0.125	4	0.152	4	0.158
5	0.142	5	0.185	5	0.192
6	0.132	6	0.172	6	0.178
7	0.131	7	0.168	7	0.175
8	0.152	8	0.192	8	0.198
9	0.139	9	0.162	9	0.168
10	0.155	10	0.179	10	0.186
11	0.146	11	0.188	11	0.195
12	0.162	12	0.171	12	0.182
13	0.135	13	0.195	13	0.201
14	0.158	14	0.166	14	0.173
15	0.149	15	0.181	15	0.190

Note: Absolute error $e_{i}$ is measured in millimeters (mm). Each component type includes 15 feature points.

**Table 11:** Statistical Results of Absolute Errors for Different Component Types.

Statistic	Flat Plate	Statistic	Free-Form Surface	Statistic	Groove
Sample Size *n*	15	Sample Size *n*	15	Sample Size *n*	15
MAE	0.151	MAE	0.174	MAE	0.181
RMSE	0.166	RMSE	0.188	RMSE	0.194
Maximum Error	0.193	Maximum Error	0.215	Maximum Error	0.218
Standard Deviation	0.018	Standard Deviation	0.022	Standard Deviation	0.020

Note: MAE = Mean Absolute Error; RMSE = Root Mean Square Error. All values are in millimeters (mm).

**Table 12:** Comparison of Positioning Accuracy for Flat Plate Components.

Method Name	MAE	RMSE	Average Processing Time
Manual Teaching Three-Point Positioning Method	6.80 mm	6.93 mm	110 s
FPFH + SAC-IA Point Cloud Matching Method	21.57 mm	22.77 mm	265 ms
TransFusion Method	0.31 mm	0.35 mm	85 ms
Proposed Method in This Paper	0.15 mm	0.17 mm	17.2 ms

**Table 13:** Comparison of Positioning Accuracy for Free-Form Surface Components.

Method Name	MAE	RMSE	Average Processing Time
Manual Teaching Three-Point Positioning Method	6.96 mm	7.10 mm	125 s
FPFH + SAC-IA Point Cloud Matching Method	22.99 mm	23.17 mm	295 ms
TransFusion Method	0.32 mm	0.36 mm	89 ms
Proposed Method in This Paper	0.17 mm	0.19 mm	17.2 ms

**Table 14:** Comparison of Positioning Accuracy for Groove Components.

Method Name	MAE	RMSE	Average Processing Time
Manual Teaching Three-Point Positioning Method	6.75 mm	6.89 mm	140 s
FPFH + SAC-IA Point Cloud Matching Method	20.26 mm	22.45 mm	315 ms
TransFusion Method	0.33 mm	0.37 mm	94 ms
Proposed Method in This Paper	0.18 mm	0.19 mm	17.2 ms

## Data Availability

The data presented in this study are available on request from the corresponding author. The data are not publicly available due to privacy restrictions.
